# Androgen receptor isoforms expression in benign prostatic hyperplasia and primary prostate cancer

**DOI:** 10.1371/journal.pone.0200613

**Published:** 2018-07-20

**Authors:** Ana Caroline Hillebrand, Lolita Schneider Pizzolato, Brasil Silva Neto, Gisele Branchini, Ilma Simoni Brum

**Affiliations:** 1 Laboratory of Endocrine and Molecular Biology, Department of Physiology, Universidade Federal do Rio Grande do Sul, Porto Alegre, Brazil; 2 Division of Urology, Hospital de Clínicas de Porto Alegre, Universidade Federal do Rio Grande do Sul, Porto Alegre, Brazil; 3 Department of Basic Sciences of Health, Universidade Federal de Ciências da Saúde de Porto Alegre, Porto Alegre, Brazil; University of Minnesota Hormel Institute, UNITED STATES

## Abstract

The role of molecular changes in the androgen receptor (AR) as AR variants (AR-Vs) is not clear in the pathophysiology of benign prostatic hyperplasia (BPH) and hormone-naïve PCa. The aim of the current work was to identify the presence of AR isoforms in benign tissue and primary PCa, and to evaluate the possible association with tumor aggressiveness and biochemical recurrence in primary PCa. The mRNA levels of full length AR (AR-FL) and AR-Vs (AR-V1, AR-V4 and AR-V7) were measured using RT-qPCR. The protein expression of AR-FL (AR-CTD and AR-NTD) and AR-V7 were evaluated by the H-Score in immunohistochemistry (IHC). All investigated mRNA targets were expressed both in BPH and PCa. AR-FL mRNA levels were similar in both groups. AR-V4 mRNA expression showed higher levels in BPH, and AR-V1 and AR-V7 mRNA expression were higher in PCa. The AR-V7 protein showed a similar H-Score in both groups, while AR-CTD and AR-NTD were higher in nuclei of epithelial cells from BPH. These results support the assumption that these constitutively active isoforms of AR are involved in the pathophysiology of primary PCa and BPH. The role of AR-Vs and their possible modulation by steroid tissue levels in distinct types of prostate tumors needs to be elucidated to help guide the best clinical management of these diseases.

## Introduction

Prostate cancer (PCa) is the most common neoplasia among Brazilian men, with 68,220 new cases estimated for the year 2018, corresponding to an estimated risk of 66.12 new cases per 100 thousand men, only behind non-melanoma skin cancer [1]. As prostatic cell growth is androgen dependent, androgen ablation therapy has become the mainstay of treatment for patients with PCa [2]. However, following a period of initial response, prostate cancer cells can acquire the ability to proliferate despite very low circulating concentrations of androgen. This androgen-independent proliferation is clinically significant, because the majority of men who develop resistance to hormonal therapy do not respond to currently available therapies. During this therapy resistance phase, the activation of androgen receptors (AR) may occur independently of hormone binding [3, 4]. It is speculated that prostate cells use different mechanisms to compensate for androgenic deprivation, and, recently, androgen receptor splice variants (AR-Vs) have been associated with that androgen-independent growth [5].

The *AR* gene is composed of eight exons encoding a 110 kD protein. The AR protein comprises different domains: an N-terminal domain (NTD), encoded by exon 1; a DNA-binding domain (DBD), encoded by exons 2 and 3; and the ligand-binding domain (LBD), encoded by exons 4 to 8, which is the region where hormone binding occurs [5, 6]. When the hormone binds to LBD, it allows the translocation of the receptor to the nucleus and the subsequent transcriptional regulation of androgen-responsive genes [7]. However, although AR-Vs lose LBD, they allow the AR signal to occur, even in the absence of androgen [5, 8–12]. Among the variants already identified, AR-V7 is the most thoroughly studied to date [5, 9, 10]. However, the role of AR-Vs in the pathophysiology of benign prostatic hyperplasia (BPH) and hormone-responsive PCa needs to be better clarified [5]. Some authors have reported the presence of isoforms in normal tissue [9–11, 13]; however, those samples were obtained from normal tissue of a prostate with cancer. It is noteworthy that samples of tissue adjacent to tumors, although morphologically confirmed as non-neoplastic tissue, can present molecular alterations like those found in malignant tissue (unpublished data). Some authors, when analyzing isoform gene expression, observed that AR-Vs are expressed at levels substantially lower than AR-FL [5, 9, 12]. In castration-resistant PCa (CRPC) samples, the AR-V expression levels are significantly higher than the samples of primary PCa, and their expression is associated with a worse clinical outcome [5, 9]. In addition, they have been associated with increased disease recurrence after radical prostatectomy compared with patients with low expression of AR-Vs [9]. The AR-Vs can have an important role in the complex regulatory mechanism of tumor cell proliferation and drive molecular changes in this tissue. AR-Vs present an interesting complex interaction between themselves. Heterodimerization between AR-Vs and with AR-FL could have important impact at cellular level [14]. As demonstrated by Zhan et al. [14], AR-V7 heterodimerizes with AR-V1, AR-V4 and AR-V6, which facilitates its nuclear localization. These isoforms could also dimerize with AR-FL, even in the absence of androgen. When dimerized to androgen-bound AR-FL, these isoforms are piggybacked into the nucleus. AR-V1 could heterodimerize with AR-V7 and as consequence the ability of AR-V7 to confer castration-resistant cell growth is inhibited, which suggest that AR-V1 could act as a negative regulator of AR-V7. Although this AR-Vs interplay were studied to investigate castration-resistant cell growth, the presence, interaction and possible consequences of AR-Vs expression should be taken in consideration even in benign tissue. The molecular mechanisms of AR-Vs interactions still unclear, especially involving cell cycle and apoptosis-related genes. To provide a better understanding of the molecular mechanisms of AR-Vs and pathophysiology of prostatic tumors, in the present work, we investigate the expression of AR-Vs, cell cycle and apoptosis-related genes in prostate tissue from patients with primary PCa or BPH, and their possible association with tumor aggressiveness and biochemical recurrence in primary PCa.

## Material and methods

### Patient selection

Tissue samples from patients with primary PCa (n = 61) or BPH (n = 128) were obtained from radical prostatectomy, prostatectomy or transurethral ressection (TURP). For mRNA analysis, 25 samples of primary PCa and 30 of BPH were used. For the immunohistochemical analysis, 6 samples of each group were used. The pathological diagnosis of the tissues was confirmed by the Pathology Service. This study was approved by the Research Ethics Committee of the Hospital de Clínicas de Porto Alegre (HCPA). Informed consent was obtained from all individual participants included in the study. Clinical data for each patient were collected from the hospital electronic records and any of the authors had access to any information which could potentially identify any individual patients.

### Gene expression analysis

Molecular analysis was performed by quantitative PCR (qPCR) following reverse transcription (RT), with a TaqMan® probe detection system (Life Technologies™) using 10-fold diluted samples. The gene for beta-2-microglobulin (*B2M*) was used as a reference gene for the stabilization of gene expression levels among the different samples. For amplification of *B2M* and *AR*, inventoried assays from Life Technologies™ were used (Hs00171172_m1 and Hs00984230_m1, respectively). It is noteworthy that the assay used for AR amplified transcripts 1 and 2 of the receptor (*AR-FL*). For the design of primers and probes specific for isoforms, the mRNA sequences described by Guo *et al*. [5], and available in UniGene under the identifications FJ235917.1 (AR-V1), FJ235919.1 (AR-V4), and FJ235916.1 (AR-V7) [5] were used. We also analyzed the cell cycle-related genes *TP53*, *MDM2*, *CDKNA* and apoptosis-related genes *BAX* and *BCL2* to study their possible association with AR-Vs and to help towards a better understanding of the molecular mechanisms of AR-Vs. The sequences of primers and probes are shown in Table 1.

**Table 1:** Sequences of primers and probes used for qRT-PCR.

Target	Sequence
*B2M*	Hs00984230_m1
*AR-FL*	Hs00171172_m1
*AR-V1*	sense 5’—AGGGTGTTTGGAGTCTCAGA—3’ antisense 5’—CCAGGAATGAATCATCTACAAA—3’ probe 5’ -TTCCTTAAAGACTACCTTCAGACTC—3’
*AR-V4*	sense 5’—GACACTAACCCCAAGCCATAC—3’ antisense 5’ -ACTGTCTGATGTTGCTCTGTG—3’ probe 5’—TTGTTTTCTGTCAGTCCCATTGGTGC—3’
*AR-V4V7*	sense 5’—CTCTTGATTGCTGACTCCCTC—3’ antisense 5’ -ACAACTACATGAGTGGTAACCA—3’ probe 5’—AGGTAGGAAAACACTATTGGTCCCGC—3’
*BAX*	Hs00180269_m1
*BCL2*	Hs00608023_m1
*CDKN1A*	Hs00355782_m1
*TP53*	Hs01034249_m1
*MDM2*	Hs00242813_m1

We designed a specific assay to amplify AR-V1, since a unique region was identified in this variant, which allowed isolated amplification of this isoform. Due to the close similarity between the cDNA sequences of isoforms AR-V4 and AR-V7, after careful analysis, the design of specific assays for these isoforms was not possible; thus, we chose the concomitant amplification of the isoforms. Therefore, we designed assays for the joint amplification of transcripts AR-V4 and AR-V7, and named this transcript AR-V4V7. The same was true for AR-V4 and AR-V3. Even though both transcripts were amplified together, to simplify understanding, we refer here the transcript AR-V3V4 as AR-V4. The ratio for AR-V4V7/AR-V4 was calculated to represent AR-V7 expression, termed here as AR-V7rv (representative value).

### Immunohistochemistry (IHC) of AR-Vs

We chose to use three different antibodies to investigate AR expression. The PG21 antibody recognizes the N-terminal domain (NTD), which is encoded by AR-FL and AR-Vs. Because of this, antibodies recognizing AR-NTD detect both AR-FL and AR-Vs. The C-19 antibody recognizes the C-terminal domain (CTD), and since LBD-truncated AR-Vs lacks the CTD region, they are consequently not recognized by the C-19 antibody. An alternative approach to detect the presence of AR-Vs is to combine data obtained using antibodies recognizing AR-NTD and AR-CTD, respectively.

IHC was performed on 4-μm sections derived from FFPE blocks using the mouse AR-V7 monoclonal antibody (Precision Antibody), diluted 1:200; the rabbit polyclonal AR (C-19) (Santa Cruz), diluted 1:400; and the rabbit polyclonal AR (PG21) (Millipore), diluted 1:400. Antigen retrieval was achieved by heating in a water bath the slides in citrate buffer (pH 6.0) for 1 hour at 95°C. Endogenous peroxidase was blocked using 3% H2O2 solution in methanol for 30 minutes. The reaction was visualized using DAB chromogen (Liquid Dab, Dako) followed by counterstaining with Harris hematoxylin. Cases were scored blind by two evaluators as clinical data using the modified H-score (histological score) method, a semiquantitative assessment of staining intensity that reflects antigen concentration [15, 16]. Briefly, the H-score was determined according to the formula: [(% of weak staining) x 1] + [(% of moderate staining) x 2] + [(% of strong staining) x 3], yielding a range from 0 to 300 [15, 16].

### Statistical analysis

Data are reported as mean (standard deviation) when parametric, and as median values with interquartile range (IQR) when non-parametric, and analyzed by Student’s *t*-test or Mann-Whitney U test, respectively. In order to verify the association between the results of isoform gene expression and the other continuous variables, a Spearman’s rank correlation was performed. All *P* values were two-sided and *P*<0.05 was considered significant. All statistical analyses were performed using SPSS 20.0 program. Graphs were constructed using SPSS or GraphPad.

## Results

### Sample characterization

Clinical characteristics of patients are described in Table 2. Among the 61 PCa samples, 40 (66.7%) showed a Gleason score ≤7(3+4), and 20 (33.3%) showed a Gleason score ≥7(4+3). One PCa case had Gleason data missing. Regarding the pathologic staging (pTNM), 4 (7.3%) were classified as T1, 44 (80.0%) were classified as T2, and 7 (12.7%) as T3. Six PCa cases had pTNM data missing. After surgery, 18 patients (32.7%) presented biochemical recurrence after a median of 53 months.

**Table 2:** Sample characterization.

	BPH	PCa	*P* value
Age at surgery, years Mean (SD)	n = 128 66.70 (8.29)	n = 61 65.85 (8.23)	0.510^a^
PSA preoperative, ng/ml Median (IQR)	n = 123 5.00 (6.54)	n = 61 7.41 (6.76)	*<0.001^b^
Estimated prostate weight ^c^, grams Median (IQR)	n = 89 62.00 (56)	n = 42 36.50 (25)	*<0.001^b^
Surgical specimen weight, grams Median (IQR)	n = 57 56.00 (35)	n = 55 42.00 (22)	*0.001^b^

a *P* value was determined T test. P values are corrected for ties

b *P* value was determined by the Mann–Whitney U test. P values are corrected for ties.

c Prostate weight estimated by ultrasound or digital rectal examination.

### AR-Vs are differentially expressed between BPH and PCa

**Fig 1:**
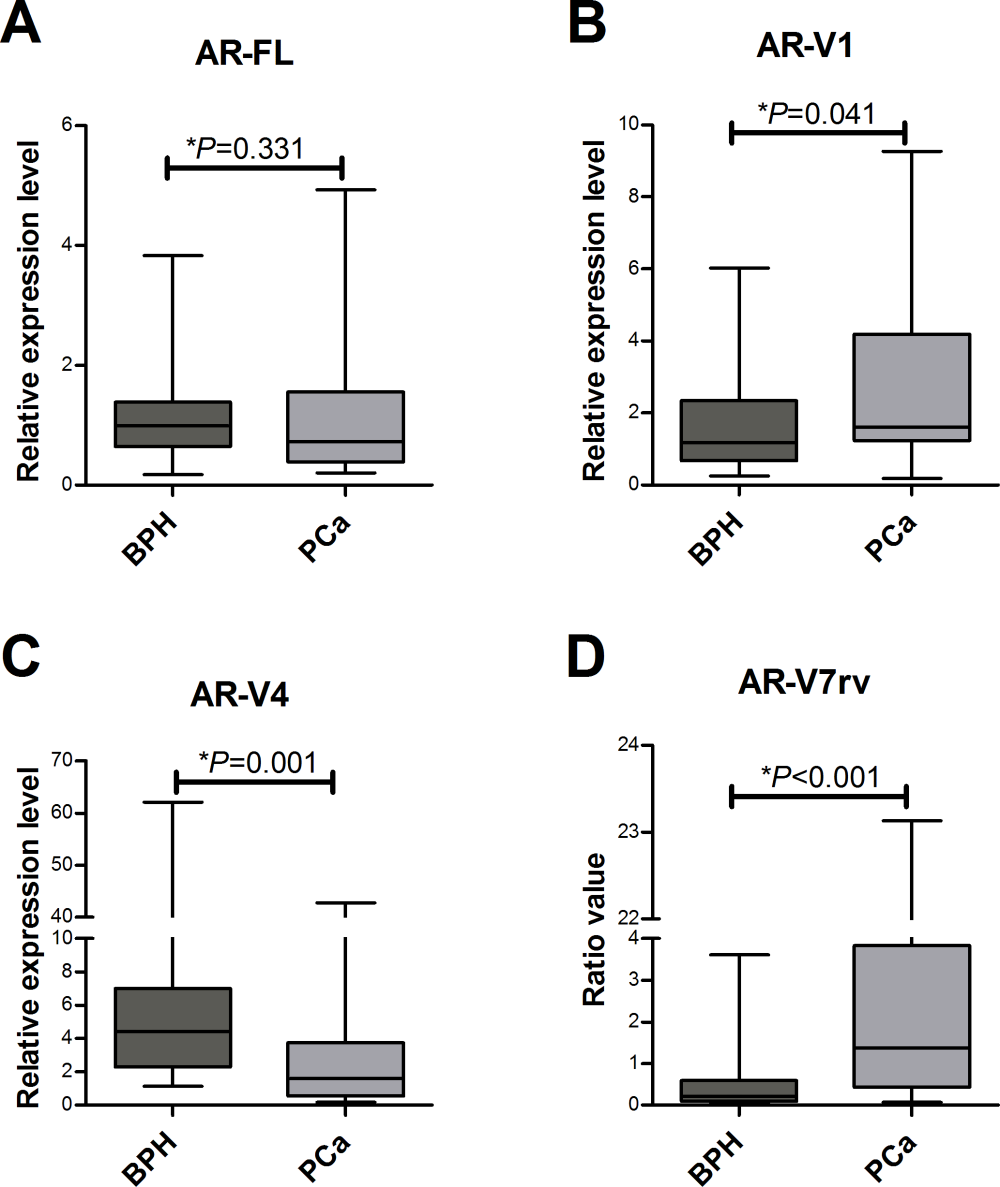
AR-Vs mRNA expression in BPH and PCa samples. All mRNA data are normalized by beta-2-microglobulin (*B2M*). For AR-FL, AR-V1 and AR-V4, values of normalized quantity means (NQM) were used to create the graphs. AR-V7rv was obtained from the ratio between AR-V4V7 and AR-V4. The bars represent the minimum to maximum values. (A) The median AR-FL expression is not significantly different comparing BPH and PCa (*P* = 0.331). (B) and (D) AR-V1 and AR-V7rv expression are higher in primary PCa than in BPH (*P* = 0.041 and *P*<0.001, respectively). (C) AR-V4 expression is significantly different comparing BPH and PCa (*P* = 0.001), showing higher levels in hyperplastic tissue. *P* value was determined by the Mann–Whitney U test.

We detected all analyzed AR transcripts in BPH and primary PCa samples. The mRNA expression of AR-FL showed no difference between the groups (*P* = 0.331) (Fig 1A). PCa samples showed higher AR-V1 gene expression compared to the BPH group (*P* = 0.041) (Fig 1B). Conversely, BPH showed higher AR-V4 gene expression compared to the PCa group (*P* = 0.001) (Fig 1C). Using the ratio of AR-V4V7/AR-V4, it was possible determine an indirect value of AR-V7, termed AR-V7rv. This was more highly expressed in PCa when compared to BPH (*P*<0.001) (Fig 1D). When PCa samples were dichotomized based on biochemical recurrence, a similar expression was observed for all mRNAs analyzed among patients who had biochemical recurrence and in those who did not present recurrence.

### The cell cycle-related and apoptosis-related genes are more expressed in PCa group

Regarding the expression of *BAX*, *BCL2*, *TP53*, *CDKN1A* and *MDM2*, all of these were more highly expressed in the PCa group (*P*≤0.001, *P* = 0.029, *P* = 0.011, *P* = 0.001, *P* = 0.001, and *P* = 0.025) than in the BPH group (Table 3).

**Table 3:** Cell cycle-related and apoptosis-related genes mRNA expression.

mRNA *median (interquartile range)*	BPH n = 27	PCa n = 26	*P* value
*BAX*	0.8660 (0.60)	2.1764 (2.64)	*0.000
*BCL2*	0.8114 (0.58)	1.2557 (2.21)	*0.029
*MDM2*	1.0992 (1.66)	3.5086 (6.50)	*0.001
*TP53*	0.9556 (1.12)	1.5580 (2.01)	*0.011
*CDK1NA*	0.6452 (0.62)	2.3317 (3.12)	*0.001

*P* value was determined by the Mann–Whitney U test. P values are corrected for ties.

* *P*< 0.05 were considered statistically significant.

### AR-NTD and AR-CTD are expressed in higher levels in the nuclei of epithelial cells from BPH samples

**Fig 2:**
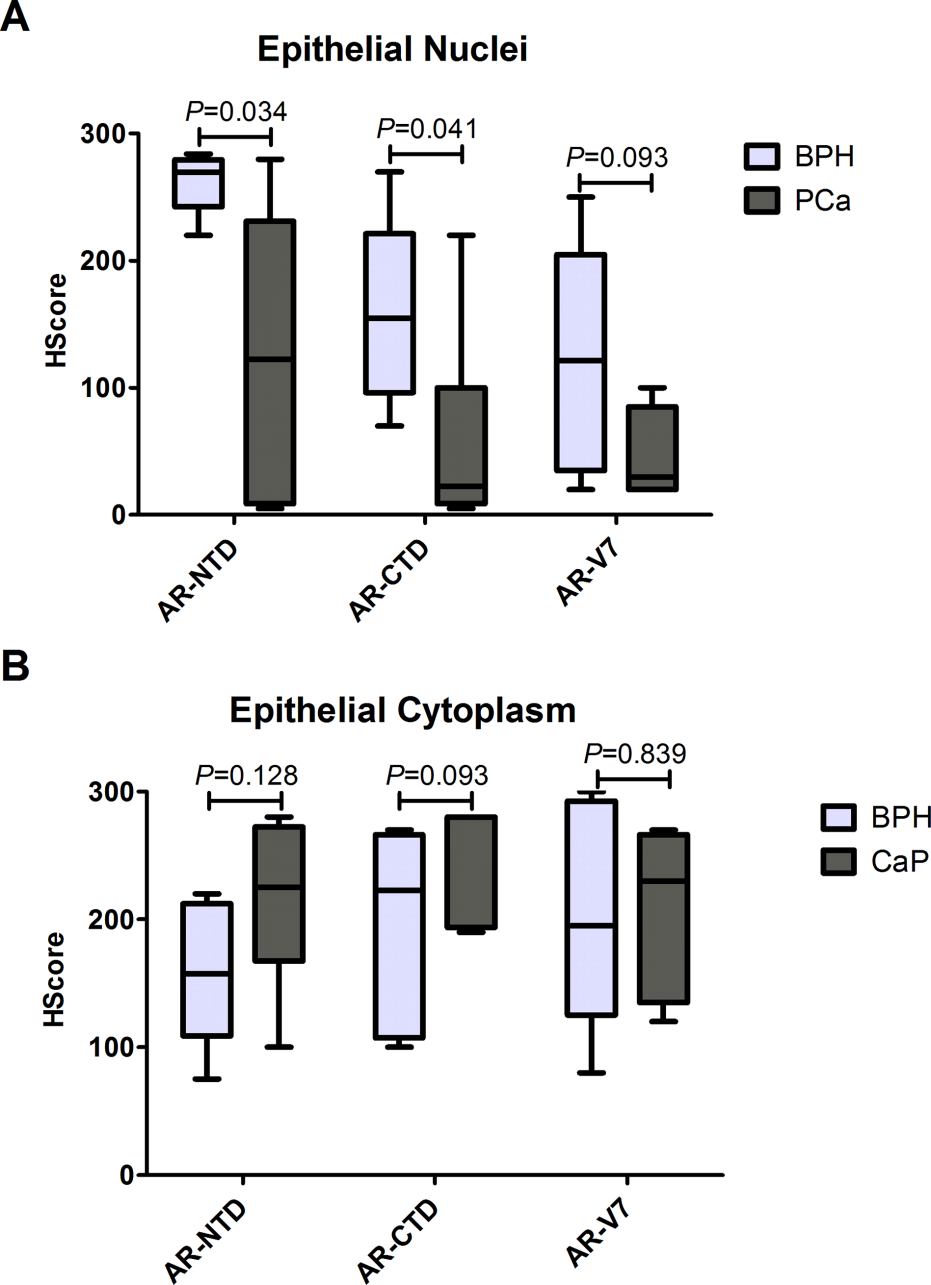
Histological score for immunohistochemical staining of AR-NTD, AR-CTD, and AR-V7. A variety of immunohistochemical staining patterns for AR-NTD, AR-CTD and AR-V7 are shown. Epithelial nuclear staining (A) for AR-NTD (PG21) and AR-CTD (C19) are higher in BPH samples than in PCa samples (*P* = 0.034 and *P* = 0.041, respectively). Although BPH group seems to have a higher AR-V7 H-Score, it was not statistically different (*P* = 0.093). Epithelial cytoplasmatic staining (B) was not different between the groups. The bars represent the minimum to maximum values.

**Fig 3:**
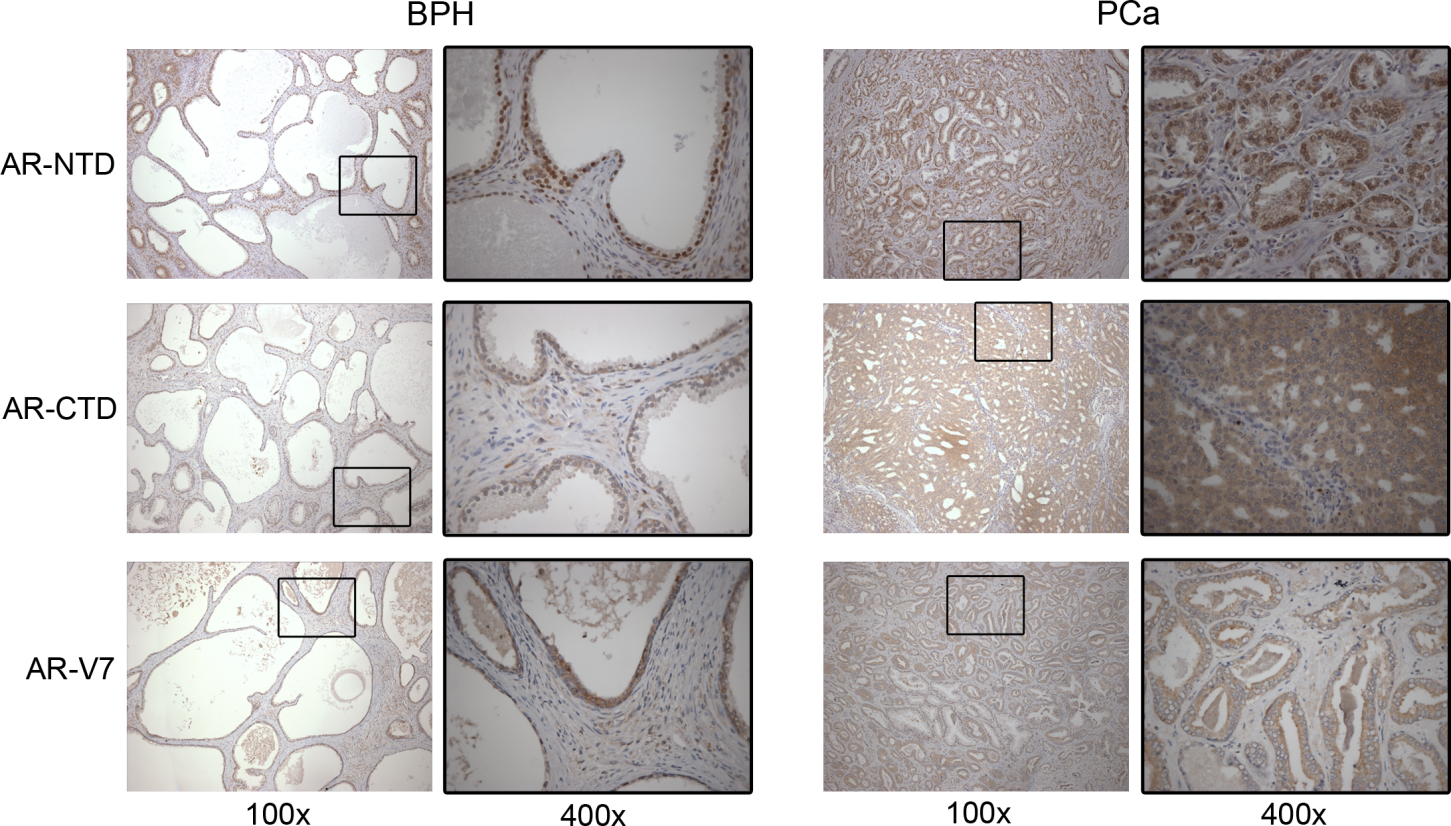
Representative images of IHC staining. 100X and 400X. For H-Score calculation we analyzed all over the slides.

We also analyzed the protein expression of AR-NTD, AR-CTD and AR-V7 by immunohistochemistry. For this analysis, the H-score (HS) was quantified separately in the nucleus and cytoplasm of epithelial and stromal compartments (Table 4, Fig 2A and 2B). AR-NTD and AR-CTD had a higher HS in epithelial nuclei of BPH group than in the PCa group (*P* = 0.034 and *P* = 0.041, respectively; Fig 2A). AR-V7 seemed to be also more highly expressed in nuclei of epithelial cells in benign tissue, but failed to reach statistical significance (*P* = 0.093, Fig 2A). Indeed, AR-V7 was not differentially expressed in any compartment, but this variant was present in epithelial cells from BPH and PCa. AR-V7 was also expressed in the cytoplasm of stromal cells at the same intensity between the groups. On the other hand, stromal nuclei were positive for AR-NTD, although stromal cytoplasm was positive for AR-V7. Fig 3 shows representative images of immunohistochemical staining on epithelial cells. When the H-score yielded a value lower than 10, results were considered negative and were represented as not detectable (ND). Using the AR-NTD/AR-CTD ratio, we observed a wide distribution in epithelial nuclei of PCa samples, although it failed to reach statistical significance (*P* = 0.818) (Figure in S1 Fig).

**Table 4:** Histological scores (H-Score) representing the histological staining patterns of AR-NTD, AR-CTD and AR-V7 protein in BPH and PCa samples.

		AR-NTD			AR-CTD			AR-V7		
		BPH	PCa	*P*	BPH	PCa	*P*	BPH	PCa	*P*
**Nuclei**	**Epithelial**	261.8 (23.8)	125.8 (116.2)	*0.034^a^	155.0 (125)	22.5 (91)	*0.041^b^	121.5 (170)	30.0 (65)	0.093 ^b^
	**Stromal**	145.0 (40)	19.5 (121)	0.065^b^	ND	ND	ND	ND	ND	ND
**Cyto-plasm**	**Epithelial**	156.7 (55.6)	215.0 (65.6)	0.128^a^	222.5 (159)	280.0 (86)	0.093 ^b^	200.0 (86.5)	209.2 (64.2)	0.839 ^a^
	**Stromal**	ND	ND	ND	25.0 (55)	ND	ND	96.7 (54.3)	75.7 (48.4)	0.495 ^a^

a Values presented as mean (SD). P value was determined by Student’s t test. P values are corrected for ties.

b Values presented as median (interquartil range). P value was determined by Mann–Whitney U test. P values are corrected for ties.

ND = Not detectable

We observed positive staining for the three antibodies in all epithelial samples, which is remarkable since benign tumors (like BPH) and primary cancer are not expected to express AR-Vs, which are supposed to arise only after androgen deprivation therapy. Nuclear stromal staining was positive only for AR-NTD in both groups. Although AR-CTD was detectable in the BPH group, the score was so minimal that it was considered negative. AR-CTD in PCa samples and AR-V7 in both nuclear stromal groups were negative.

Using Spearman’s rank correlation analysis, we found a positive correlation among AR-Vs (Table 5). In the PCa group, AR-FL was positively correlated with *BCL2* (0.464, *P* = 0.030; Table 6). In the BPH group, AR-V1 and AR-V4 were positively correlated with prostate weight (0.477, *P* = 0.025; and 0.711, *P*<0.001; Table 5). The positive correlation between AR-V4 and prostate weight is not surprising given that BPH (which presented a higher prostate weight) has a higher mRNA level of this isoform when compared with PCa. Although AR-V1 was more highly expressed in PCa, this isoform showed a positive correlation with prostate weight in the BPH group. In addition, the ratio of *BCL2*/*BAX* showed a regular negative correlation (-0.413, *P* = 0.036; Table 6) with the surgical specimen weight. In PCa, we found a regular positive correlation between AR-V4 and Gleason score (0.493, *P* = 0.0012), suggesting that with the increase of tumoral aggressivity, there was an increase of AR-V4 expression.

**Table 5:** Correlation coefficients between AR-Vs and clinical features.

		BPH *r*_*s*_ (*P*)	PCa *r*_*s*_ (*P*)
Correlation between AR-Vs			
AR-V1	AR-V4	0.387 (0.035)	0.457 (0.022)
	AR-V7rv	0.394 (0.031)	-
Correlation between AR-Vs and clinical features			
AR-V1	Estimated prostate weight	0.477 (0.025)	-
AR-V4	Estimated prostate weight	0.711 (<0.001)	-
	Gleason	-	0.493 (0.012)
Correlation between ratios and clinical features			
AR-FL/AR-V1	Age at surgery	-0.364 (0.048)	-
AR-FL/AR-V4	Age at surgery	-0.405 (0.027)	-
	Estimated prostate weight	-0.495 (0.019)	-
	Gleason	-	-0.475 (0.016)

Spearman correlation for association between variables. The results are presented as *r*_*s*_ (*P*). Values of *P*< 0.05 were considered statistically significant. Only statistically significant correlations are showed.

**Table 6:** Correlation coefficients between cell cycle-related and apoptosis-related genes and other variables.

		BPH *r*_*s*_ (*P*)	PCa *r*_*s*_ (*P*)
Correlation between cell cycle-related and apoptosis-related genes and AR-Vs			
*BCL2*	AR-FL	-	0.464 (0.020)
	AR-V7rv	-	0.450 (0.024)
*MDM2*	AR-FL	-	0.434 (0.030)
	AR-V1	0.422 (0.028)	0.549 (0.004)
Correlation between apoptosis-related genes and ratio			
*BCL2*	AR-FL/AR-V4	-	0.472 (0.017)
Correlation between cell cycle-related and apoptosis-related genes and clinical features			
*TP53*	PSA	-	-0.439 (0.025)
*BCL2*/*BAX*	Surgical specimen weight	-	-0.413 (0.036)

Spearman correlation for association between variables. The results are presented as *r*_*s*_ (*P*). Only statistically significant correlations are showed. Values of *P*< 0.05 were considered statistically significant.

Age at surgery was negatively correlated with the ratios AR-FL/AR-V1 (-0.364, *P* = 0.048), AR-FL/AR-V4 (-0.405, *P* = 0.027) and AR-FL/AR-V4V7 (-0.377, *P* = 0.040) in the BPH group. These associations between ratios and age were not found in the PCa group, but in this group, the ratios AR-FL/AR-V4 and AR-V7rv were positively correlated with *BAX* (0.472, *P* = 0.017; and 0.450, *P* = 0.024).

Besides the correlation between cell cycle-related and apoptosis-related genes and AR-Vs and clinical features, these genes were correlated among themselves. The correlations of cell cycle-related genes (*TP53*, *MDM2*, *CDKN1A*) and apoptosis-related genes (*BAX*, *BCL2* and ratio *BCL2*/*BAX*) are presented in Table 6.

### High grade versus low grade PCa

**Fig 4:**
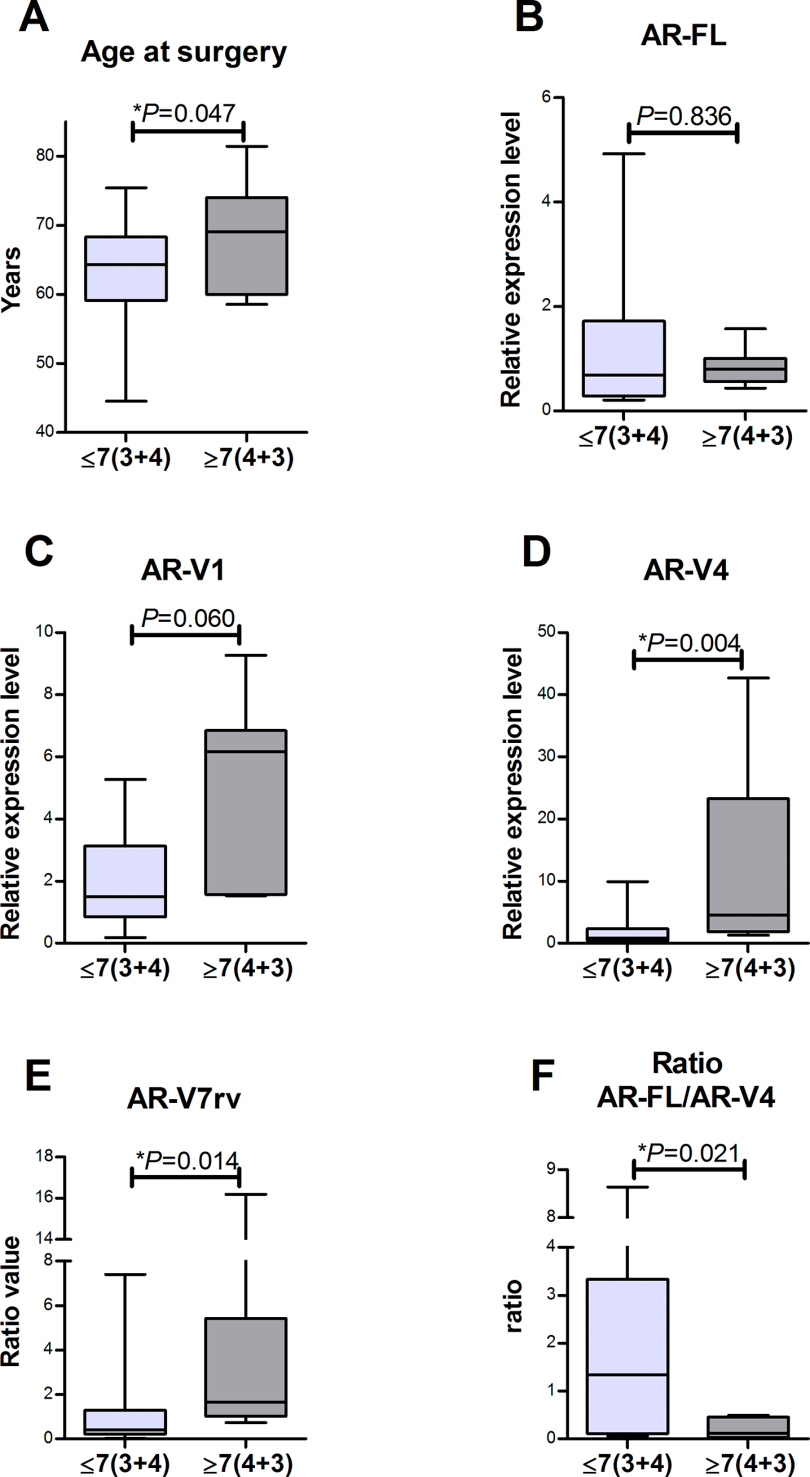
Stratification of PCa samples between Gleason grade (≤7(3+4) and ≥7(4+3). (A) Age at surgery. The group low grade (≤7(3+4) was younger than the high grade group (≥7(4+3) (63 and 68 years, respectively. *P* = 0.047). (B) AR-FL levels were similar between groups (*P* = 0.836). (C) AR-V1 levels failed to reach significance (*P* = 0.060), but seems to be up-regulated in high grade group (≥7(4+3). (D) and (E) AR-V4 and AR-V7rv levels are higher in high grade group (≥7(4+3) (*P* = 0.004 and *P* = 0.014). (F) AR-FL/AR-V4 ratio was higher in low grade group (≤7(3+4), *P* = 0.021). The bars represent the minimum to maximum values.

When primary PCa samples were stratified for Gleason score (≤7(3+4) and ≥7(4+3), high-grade patients were older (*P* = 0.047, Fig 4A) and had higher AR-V4 levels (*P* = 0.004, Fig 4D) and higher AR-V7rv (*P* = 0.014, Fig 4E) when compared with the low-grade group. Although AR-V1 levels did not show statistical significance (*P* = 0.060, Fig 4C), this variant had a wide distribution in the high-grade group (≥7(4+3), which is in concordance with our results from other isoforms. AR-FL levels (*P* = 0.836, Fig 4B) were similar between low and high grades. The ratio of AR-FL/AR-V4 was higher in the low-grade samples (*P* = 0.021, Fig 4F). Regarding the biochemical relapse, the mean time until relapse was 53 months. In high-grade PCa, this time was significantly lower (31 months) than low-grade PCa (65 months) (*P* = 0.004), represented by the Kaplan-Meyer curve (Figure in S2 Fig).

In a Cox regression univariable model (Table in S1 Table), the ratio of AR-V1/AR-FL was associated with a higher risk of biochemical recurrence (HR = 1.172, *P* = 0.045) and *BLC2*/*BAX* was associated with protection of biochemical recurrence (HR = 0.123, *P* = 0.009). These associations remained when these variables were tested together in a multivariable model, (HR = 1.219, *P* = 0.038; and HR = 0.104, *P* = 0.014; respectively).

## Discussion

We investigated the AR-FL expression using an assay specific for transcripts 1 and 2 (GI:349501065 and GI:349510166), which prevents untrue conclusions about differences in AR-FL and AR-V expression. We also analyzed AR-V mRNA, and importantly, we detected the expression of all isoforms in samples from hyperplastic tissue and primary PCa. Truncated AR-Vs were previously assumed to be expressed primarily in CRPC, where, at least for AR-V7, their presence was associated with resistance to therapy [17, 18]. This is a remarkable finding of this work, since we evaluated tissue from hormone-naïve PCa, while others have mostly evaluated advanced cancer, castration-resistant cancer, metastasis sites or even cell lines [5, 8, 9, 11, 12, 17–23]. Our detection of AR-Vs in BPH complements the data reported in the literature, which demonstrate the expression of these isoforms in benign tissue from radical prostatectomy for cancer removal, malignant tissue and metastatic sites [5, 8, 9, 11, 12, 18, 22]. This wide distribution was recently confirmed by a whole transcriptome analysis by The Cancer Genome Atlas (TCGA), which demonstrated that AR-V7 is the most abundant AR-V expressed in human cell lines, human xenografts and clinical samples [21]. Our finding that AR-Vs are expressed in hormone-naïve primary PCa is notable, and is in concordance with the TCGA study [21]. The data of gene expression showed a large range of variation in both groups, which was also observed in previous studies [19]. We also found a correlation among the AR-Vs, which was expected since all isoforms are derived from the same pre-mRNA. The formation of one or other isoform occurs by mechanisms not precisely described until now, but it is expected that the resulting molecules are correlated at least to some level.

Together, our findings suggest that these constitutively active isoforms participate in different proliferative events in different types of prostatic tumors, not only as the main driver of castration resistance. During tumor progression, selection for cells that express AR-Vs could occur, via selection of more malignant or castration-resistant subclones or even via the hormone-dependant regulation of AR isoforms [12]. It has been already demonstrated that AR isoforms show weak expression in androgen-dependent cell lines (LNCaP and LAPC4), while in androgen-independent lines, such as CWR22Rv1 and those derived from LNCaP (C81, C4-2 and C4-2B), the expression of the AR isoforms is significantly higher [5]. These data imply an inverse correlation between AR isoform expression and the dependence of androgens [5]. It has been demonstrated *in vitro* by Watson et al. [12] that AR-FL and AR-V (AR-V1 and AR-V7) expressions are upregulated by castration, while re-administration of androgens suppressed their expression. In the present study, PCa showed high AR-V1 and AR-V7rv expression, while steroid (testosterone, 4-androstenedione, progesterone) expression was decreased (unpublished data).

When the PCa group was stratified into Gleason ≤7(3+4) and ≥7(4+3), age was higher in patients with Gleason ≥7(4+3). This indicates that older patients tend to present more aggressive PCa than younger patients. Despite the relatively small cohort number, a greater AR-V expression was seen in PCa samples with a high-grade Gleason score compared to a low-grade Gleason score. AR-V4 and AR-V7rv levels were higher in the high-grade ones. Even without statistical significance, AR-V1 showed a higher distribution in high-grade tumors. In line with these results, we found an inverse correlation between the AR-FL/AR-V4 ratio and the Gleason score, which indicate that AR-V expression increases with higher Gleason scores.

When we stratified PCa cases by whether biochemical relapse occurred or not, we observed a similar expression of all mRNAs analyzed between those patients who had biochemical recurrence and in those who did not present recurrence, which is in concordance with the results reported by Zhao *et al*. [19]. They showed that neither the AR-V7 nor its negative regulator AR-V1 were associated with biochemical recurrence in a cohort of men at indeterminate risk of progression [19].

Some studies have reported a positive correlation between AR-V7 and AR-V1 in PCa samples [18, 19], in agreement with our data. Interestingly, Watson *et al*. [12] observed that AR-V1 and AR-V7 co-expression results in complete avoidance of the gain of function conferred by AR-V7, indicating that AR-V1 seems to play a negative role on AR-V7, which was recently confirmed and well discussed by Zhan [14]. The higher expression of AR-V1 in carcinoma samples compared with BPH samples seems to be very promising data, although it must be confirmed with a larger number of patients. AR-V1 blocks the ability of AR-V7 to confer castration-resistant cell growth, and when these isoforms are co-expressed, AR-V7 does not transactivate its targets [14]. AR-V1 has an ability to selectively activate a canonical AR-FL signal. Thus, if both isoforms are expressed in both tissues, with PCa presenting higher levels of AR-V1, it probably directs the canonical AR signal as the main pathway in primary and androgen-responsive PCa. On the other hand, AR-V4, which is more highly expressed in BPH, transactivates both canonical AR-targets and AR-V-specific targets. To our understanding, this is an interesting finding of our work, since in this benign tissue, both AR-V7 and AR-FL pathways could be activated, showing an absence of a trend to either route. As previously described, advanced and aggressive PCa samples have a higher AR-V7 expression [9, 18], which was confirmed by our work when samples were dichotomized based on their Gleason scores. In this case, it is possible that AR-V7 levels surpass AR-V1 levels, favoring the “AR-V7-pathway”, which was already reported in CRPC and metastasis [5, 8, 9, 11, 12, 18, 20, 23]. The presence, interaction and possible consequences of AR-Vs expression should be taken in consideration also in benign tissue. In summary, BPH have more AR-V4 (which could activate both AR-V7 and AR-FL pathways), primary PCa have more AR-V1 (to activate AR-FL canonical targets and inhibit AR-V7), and CRPC, according to the literature, have more AR-V7 (castration-resistant cell growth driver). Unfortunately, in this work we did not have access to CRPC biopsy samples. In addition, the strong positive correlation between AR-V4 and Gleason score emphasized the role of AR-V4 in PCa progression, which is also indicated by the positive correlation between prostate weight with AR-V1 and AR-V4 expression. In high-grade PCa, AR-V4, which could activate both AR-V canonical-targets and AR-FL canonical-targets, could enable the activation of both sides of the AR pathway, making it possible that the cell takes advantage of both pathways.

The correlation between *BCL2* with AR-FL and AR-V7rv in PCa suggests an association between the AR-mediated pathways and the antiapoptotic process involved in tumor development. Elevation in the level of the BCL2 protein has been shown to provide protection from apoptosis, and the *BCL2* gene family is implicated in the development of CRPC and resistance to therapy since its expression increases during progression of prostate cancers [24, 25]. These findings corroborate the hypothesis that AR and AR-V7 have a role in the mechanisms of proliferation and apoptosis, and may contribute to cancer progression. *MDM2* was also positively correlated with AR-FL in PCa, and with AR-V1 in PCa and BPH. However, the interactions among AR, AR-Vs and *MDM2* are poorly described. One study suggests that *MDM2* induces AR ubiquitination, promoting its degradation [26]. Therefore, the association between cell cycle-related and apoptosis-related genes and AR-Vs suggests new ways to help understand the development of primary PCa.

We used IHC to investigate protein expression in FFPE samples from BPH and primary PCa. This technique is reliable and widely used in clinical diagnosis. Our approach to detect protein expression was based on three antibodies: AR-NTD (which recognizes AR-FL and AR-Vs), AR-CTD (which recognizes AR-FL) and AR-V7 (specific to this isoform). Using two antibodies against different regions of the AR protein (AR-NTD and AR-CTD), it is possible to successfully show the overall frequency of C-terminal truncated AR-Vs [27]. We observed a wide distribution of the AR-NTD/AR-CTD ratio in epithelial nuclei of PCa samples. This finding suggests the presence of AR-Vs transcripts that lack part or all of the C-terminus, which is in line with previous work [27]. To detect AR-V7, we used a specific antibody against this isoform, rather than the alternative approach to detect all truncated variants suggested by Zhang et al. [27].

We observed considerable heterogeneity in AR staining within samples. Epithelial cells showed positive staining for the three antibodies in both groups. AR-NTD and AR-CTD had a higher H-score (HS) in epithelial nuclei of BPH. AR-NTD was stained less intensively and more heterogeneously in epithelial nuclei and stromal cells of PCa than in BPH, and this is consistent with the report of Miyamoto [28]. AR-V7 showed the same trend, despite it failed to reach statistical significance. It is important to note that this isoform was detected in epithelial cells from BPH and PCa, and in stromal cytoplasm, but at lower levels. In addition, stromal nuclei were positive only for AR-NTD, while stromal cytoplasm was positive for AR-V7. This positive stromal staining is remarkable, due to the importance of stromal AR in the development of normal prostate and BPH, and also in the growth and progression of PCa [29]. Of note, positive staining observed with AR-NTD antibody may reflect the expression of other variants than AR-FL and AR-V7 (like AR-V1, AR-V4).

AR-V7 protein expression was similar between the analyzed groups. Our results differ from Guo [5], whose immunohistochemistry analysis on human prostate tissue revealed higher levels of AR-V7 (AR3) in malignant prostate tissues compared with their benign counterparts. An interesting finding of this author was a remarkable redistribution in AR-V7 cellular localization, showing high nuclear staining in hormone-resistant tumor samples compared with their hormone-naïve counterparts, showing that the nuclear translocation of AR-V7 is significantly increased in hormone-resistant tumors [5]. It is important to note that although our results are different, we demonstrate both AR-FL and AR-V7 expression in epithelial and stromal compartments. Although AR-V7 is truncated after exon 3 and lacks the complete nuclear localization sequence, it has been identified in the nucleus of epithelial cells [5, 16, 20, 23].

For AR action, nuclear import is an essential step. Different AR-Vs have different abilities to translocate to the nucleus. For example, AR-V7 is expressed predominantly in the nucleus when alone, but other isoforms, like AR-V1 and AR-V4, mainly localize in the cytoplasm [14]. As is already known, nuclear import of AR requires the dimerization of the receptor, which is also essential for AR-Vs. AR-V1, V4 and V6 heterodimerize with AR-V7, which facilitates their nuclear localization. These AR-Vs can also heterodimerize with AR-FL (in presence or absence of androgens), but their subcellular localization is not affected in androgen-deprived conditions. When AR-FL is bound to an androgen, the isoforms are piggybacked into the nucleus. This AR-V–AR-FL interaction can mitigate the ability of enzalutamide to inhibit androgen-induced AR-FL nuclear localization [14]. AR-V7 is the most abundant AR-V in clinical specimens, and its role in mediating castration resistance is sustained by clinical evidence. The data presented by Zhan [14] indicates that besides its transcriptional activity, AR-V7 could activate both AR-FL and other AR-Vs, which could be an important mechanism of action. Although many AR-Vs localize mainly in the cytoplasm when expressed alone, their clinical relevance should not be ignored. Since these AR-Vs are expressed together with AR-V7, they could contribute to the development of resistance, possibly adding their functions to the canonical transcriptional program of AR-FL. Nonetheless, this does not mean all AR-Vs have a synergistic action; indeed, AR-V1 could block the capability of AR-V7 to induce castration-resistant proliferation [12, 14].

The use of AR-V7 expression has been proposed as a biomarker for PCa relapse [16, 20] and even as a survival marker [20]. One of the proposed mechanisms of AR-V7 activity is that it could activate AR-FL in the absence of hormones, explaining how cancer cells could use AR-V7 as an escape from therapy [20]. Previous data has suggested that AR-V expression increases as patients progress to malignant prostate adenocarcinoma, being a consequence of therapy and a driver of therapy resistance [5]. In line with this theory, we believe that during the evolution to hormone-independent, metastatic disease, prostate adenocarcinomas may therefore express progressively more AR-Vs. Additionally, these results corroborate evidence shown in multiple studies from independent laboratories, which demonstrates that AR-V expression correlates with the development of ADT resistance in numerous model systems and clinical samples [5, 8, 11, 14, 17, 18].

However, it is noteworthy that AR-Vs are also expressed in benign tissue from BPH and in primary PCa. All samples used in our research were obtained from patients who had never undergone ADT, so our data do not support the suggestion of variants arising only after therapy, as a response to the treatment. The current idea is that the presence of AR-Vs is also biologically relevant in BPH and primary PCa. In addition, studies to help elucidate the role of AR-Vs in benign tissue and hormone-naïve PCa are needed to better clarify the involvement of these isoforms in the pathophysiology of prostatic tumors.

## Conclusions

These results support the assumption that the constitutively active AR-Vs are involved in the pathophysiology of prostatic tumors. Our data shows that the AR-Vs participate in different proliferative events in prostate cells, yet the exact role of these isoforms in benign tissue and primary cancer needs to be elucidated. The identification and functional characterization of differentially expressed molecules between normal and tumoral tissues are fundamental steps to achieving a better understanding not only of the carcinogenic process and the development of new anti-tumoral strategies, but also a better understanding of the processes that govern proliferation in benign tissues such as BPH. Additional studies aiming to elucidate in vitro hormonal modulation of AR-Vs may contribute to the understanding of their role in prostate physiology and tumor development and progress.

## Supporting information

S1 Fig Ratio AR-NTD/AR-CTD in nuclei of epithelial cells. Distribution of the ratio AR-NTD/AR-CTD in epithelial nuclei of samples from BPH and PCa (*P* = 0.818).
(TIF)

S2 Fig Kapplan-Meier curve for biochemical recurrence in PCa. PCa samples were stratified for Gleason score ≤7(3+4) and ≥7(4+3). Samples with Gleason ≥7(4+3), time to recurrence was significantly lower (31 months) than samples with Gleason ≤7(3+4) (65 months) (*P* = 0.004).
(PDF)

S1 Table Cox regression between variables. Ratio AR-V1/AR-FL was associated with a higher risk of biochemical recurrence (HR = 1.172, *P* = 0.045) and *BLC2*/*BAX* was associated with protection of biochemical recurrence (HR = 0.123, *P* = 0.009). In the multivariable model these associations remained when these variables were tested together (HR = 1.219, *P* = 0.038; and HR = 0.104, *P* = 0.014; respectively).
(PDF)
