# Supplementary figures and images for: Androgen receptor isoforms expression in benign prostatic hyperplasia and primary prostate cancer
Source: PLoS One. 2018 Jul 20;13(7):e0200613. doi: 10.1371/journal.pone.0200613 (PMC6054396; doi:10.1371/journal.pone.0200613)

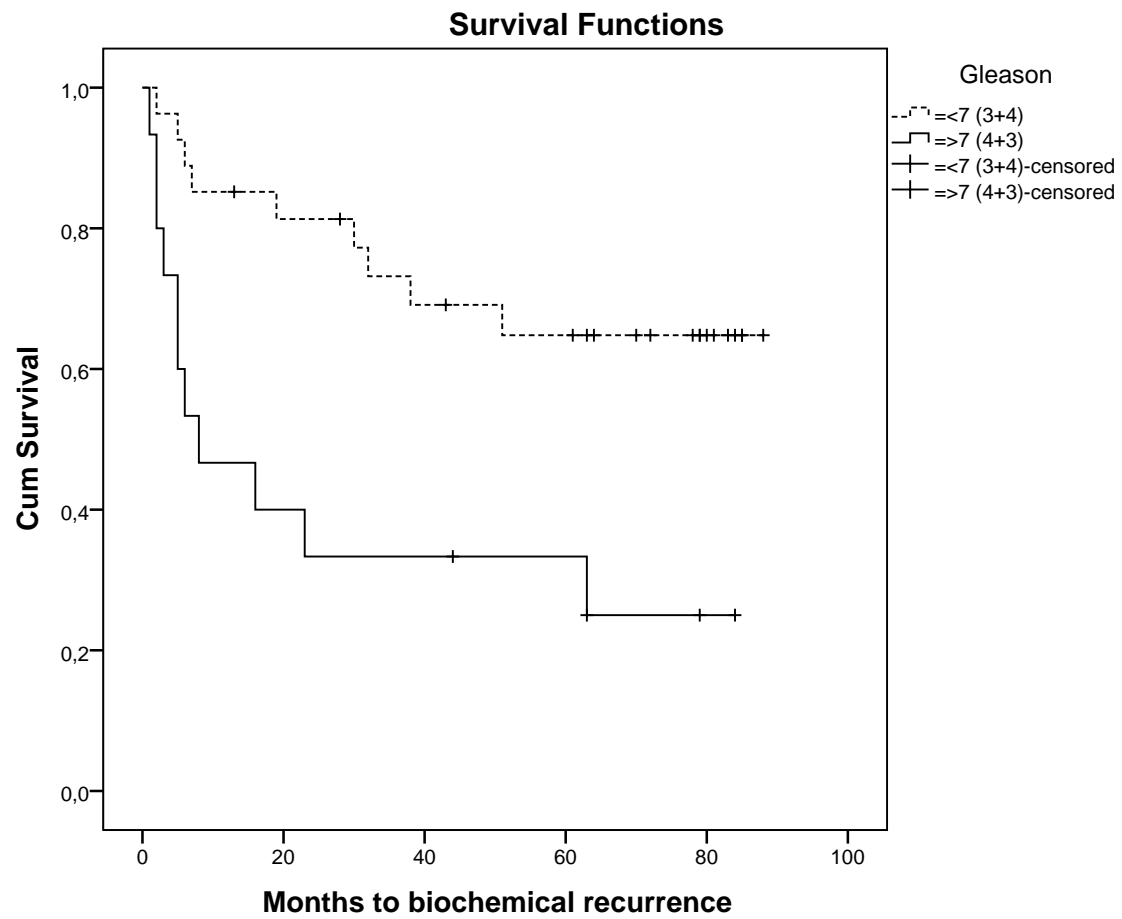

Supplement: S2 Fig — PCa samples were stratified for Gleason score ≤7(3+4) and ≥7(4+3). Samples with Gleason ≥7(4+3), time to recurrence was significantly lower (31 months) than samples with Gleason ≤7(3+4) (65 months) (P = 0.004). (PDF) [file pone.0200613.s002.pdf]
