# Supplementary material for: Androgen receptor isoforms expression in benign prostatic hyperplasia and primary prostate cancer
Source: PLoS One. 2018 Jul 20;13(7):e0200613. doi: 10.1371/journal.pone.0200613 (PMC6054396; doi:10.1371/journal.pone.0200613)
Supplement: S1 Table — Ratio AR-V1/AR-FL was associated with a higher risk of biochemical recurrence (HR = 1.172, P = 0.045) and BLC2/BAX was associated with protection of biochemical recurrence (HR = 0.123, P = 0.009). In the multivariable model these associations remained when these variables were tested together (HR = 1.219, P = 0.038; and HR = 0.104, P = 0.014; respectively). (PDF) [file pone.0200613.s003.pdf]

S1Table. Cox regression between variables.

COX Regression - Modelling												
	Univariable				Multivariable1				Multivariable2			
	HR	CI 95%		*P*	HR	CI 95%		*P*	HR	CI 95%		*P*
**AR-FL**	0,838	0,406	1,728	0.632								
**AR-V1**	1,205	0,943	1,539	0.137	1,359	1,056	1,748	0.017				
**AR-V4**	1,008	0,942	1,078	0.827								
**AR-V7rv**	0,907	0,703	1,169	0.450								
**AR-V4V7**	1,045	0,837	1,305	0.699								
**AR-FL/AR-V1**	0,403	0,095	1,699	0.216								
**AR-FL/AR-V4**	0,833	0,573	1,211	0.339								
**AR-FL/AR-V7**	0,417	0,082	2,125	0.293								
**AR-V7/AR-FL**	1,006	0,91	1,113	0.901								
**AR-V7/AR-V1**	0,461	0,185	1,147	0.096								
**AR-V4/AR-FL**	1,006	0,946	1,07	0.846								
**AR-V4/AR-V1**	0,968	0,799	1,174	0.742								
**AR-V4/AR-V7**	1,015	0,858	1,202	0.861								
**AR-V1/AR-FL**	**1,172**	**1,003**	**1,369**	**0.045**					**1,219**	**1,011**	**1,469**	**0.038**
**AR-V1/AR-V4**	0,949	0,718	1,255	0.714								
**AR-V1/AR-V7**	0,996	0,509	1,948	0.991								
**BAX**	1,114	0,892	1,391	0.341								
**BCL2**	0,629	0,329	1,2	0.159	0,632	0,329	1,216	0.169				
**BCL2/BAX**	**0,123**	**0,025**	**0,591**	**0.009**					**0,104**	**0,017**	**0,629**	**0.014**
**TP53**	1,119	0,912	1,374	0.282								
**CDKN1A**	0,842	0,596	1,19	0.329								
**MDM2**	1,003	0,912	1,104	0.945								

Outcome: Time to relapse
Adjusted by age.
Multivariable 1: variables AR-V1 and BCL2 adjusted by age.
Multivariable 2: ratios AR-V1/AR-FL and BLC2/BAX adjusted by age.
